# Analgesic and anti-inflammatory potential of Lupeol isolated from Indian traditional medicinal plant *Crateva adansonii* screened through in vivo and in silico approaches

**DOI:** 10.1186/s43141-021-00167-6

**Published:** 2021-05-04

**Authors:** Thirumalaisamy Rathinavel, Subramanian Ammashi, Gnanendra Shanmugam

**Affiliations:** 1 Department of Biotechnology, Sona College of Arts and Science, Salem, Tamil Nadu 636 005 India; 2 Department of Biochemistry, Rajah Serfoji Government College (Autonomous), Thanjavur, Tamil Nadu 613 005 India; 3 Department of Biotechnology, Yeungnam University, Gyeongsan, South Korea

**Keywords:** Analgesic, Anti-inflammatory, *Crateva adansonii*, Lupeol

## Abstract

**Background:**

Lupeol, a triterpene bioactive compound isolated from Indian traditional plant *Crateva adansonii* acted as promising and alternative anti-inflammatory agent to treatments of diseases related to inflammation. The inflammatory process in the body serves an important function in the control and repair of injury. However, it is self-perpetuating in number of disease conditions, which must be prevented and treated. Worldwide most prescribing NASID drug shows severe side effects. Whereas drug from natural origin shows dual inhibition of inflammatory and analgesic target protein with more efficacy and less side effects than NSAID drugs. Our study aims to isolate and screen the analgesic and anti-inflammatory potential of lupeol, a pentacyclic triterpenoid isolated from leaf extract of *Crateva adansonii* belongs to *Capparaceae* family commonly used Indian traditional medicine for treating inflammatory diseases.

**Results:**

Methanol and chloroform leaf extracts (ME and CE) and lupeol fraction (LF) of plant *Crateva adansonii* is investigated through employing in vivo male Wistar albino rat model. Acute toxicity study of *C*. *adansonii* ME and CE leaf extracts reveals that no mortality and no behavioral changes in experimental animals up to 2 g/kg. So no lethal dose we consider two optimal doses 200 and 400 mg of plant leaf extracts for in vivo inflammatory and analgesic study. In vivo acute and chronic anti-inflammatory activity was carried out through carrageenan-induced rat paw edema and cotton pellet-induced granuloma models. LF (100 mg/kg, oral route) of *Crateva adansonii* evoked highest percentage of inflammation inhibition (50 and 33.96% respectively) in both in vivo acute and chronic inflammation model among all tested samples (ME and CE 200 mg and 400 mg/kg, oral route) including reference standard (10 mg/kg, oral route) indomethacin. Carrageenan-challenged experimental animals were screened for one inflammatory marker enzyme myeloperoxidase (MPO), inflammatory products such as Prostaglandrin E_2_ (PGE_2_), and eight different cytokines markers (TNFα, IL-6, IFN γ, IL-1α, IL-1β, MCP-1, Rantes, and MIP) associated with inflammation reveals that LF (100 mg/kg, oral route) of *Crateva adansonii* shows prominent anti-inflammatory activity than reference standard indomethacin (10 mg/kg, oral route) over all these biological tested parameters. In vivo analgesic assays such as hot plate assay and acetic acid-induced writhing assay revealed that LF (100 mg/kg, oral route) possesses significant analgesic activity (11.60 s and 69.05%) when compared with standard drug pentazocine(10 mg/kg, oral route). Finally, we made an in silico screening of lupeol against analgesic (nAChR) and anti-inflammatory (COX-2) target proteins reveals that lupeol possess highest binding affinity with nAChR and COX-2 target proteins (− 8.5 and − 9.0 Kcal/mol) over the reference standard pentazocine and indomethacin (− 7.0 and − 8.4 Kcal/mol) respectively.

**Conclusion:**

The present study result provides a pharmacological evidences for analgesic and anti-inflammatory potential of lupeol isolated from Indian traditional plant *Crateva adansonii* act as a multi-target agent with immense anti-inflammatory potential targeting key molecules of inflammation such as MPO, PGE_2_, and eight pro-inflammatory cytokine markers. Outcome of present study is to find promising anti-inflammatory bioactive agents from the cheapest Indian traditional medicinal plant sources useful for pharmaceutical industries.

## Background

Prime role of inflammation is to resolve the noxious stimuli such as pathogens, damaged cells, toxic compounds, or irradiation through body immune response and contribute tissue homeostasis [1], and store the inflamed cells through the healing process [2]. Inflammation is therefore considered as a protection mechanism to the body cells against such noxious stimuli [3]. This mitigation process depends on the delicate balance between pro-inflammatory and anti-inflammatory mediators secreted during inflammatory immune response. However, some mediators such as interleukin (IL)-12 possess both pro- and anti-inflammatory properties [4].

Anti-inflammatory key molecule involved in modulating inflammatory immune response is that transcriptional factor nuclear factor κ B (NF κ B) play prime role in activating and synthesizing numerous pro-inflammatory cytokines such as IL-1β, IL-6, and TNF-α and leads to tissue injury. NF-κB is found active in most of the inflammatory disease conditions such as inflammatory bowel disease, arthritis, sepsis, gastritis, asthma, and atherosclerosis [5]. In some disease conditions, NF-κB activators are produced which will cause inflammation of particular organ, i.e., elevation of osteoprotegerin causes over expression of NF-κB leads to cardiovascular disease and mortality [6]. NF-κB signaling controls gene expression of cyclooxygenases (COXs) for the decrease in prostaglandin (PG) production which consequently reduces pain and inflammation.

Anti-pyretic potential of non-steroidal anti-inflammatory drugs (NSAIDs) are widely prescribed for analgesic and anti-inflammatory treatments. Chronic usage of NSAIDs causes major toxicity risk associated with renal failure and gastrointestinal problems. Medicinal plants contain promising wide range of compounds with different pharmaceutical activities [7]. Medicines from herbals are considered as valuable resource due to their low cost and lesser adverse effects [8, 9]. Numerous traditional medicinal plants and their phytocompounds are potent anti-inflammatory agents to treat various diseases linked with inflammation [10, 11].

*Crateva adansonii* is commonly known as garlic pear tree that belongs to *Capparidaceae* family. Leaves are trifoliate and flowers are white or cream color, bark is grey, and wood is yellowish-white in color. It is a moderate-sized deciduous tree distributed throughout India [12]. The plant parts such as leaf and bark were widely used as traditional medicine to treat many inflammatory diseases conditions. *Crateva adansonii* leaf extract also inhibit enzyme xanthine oxidase responsible for inflammatory arthritis [13]. Our previous study on phytocompound lupeol isolated from chloroform leaf extract of Indian traditional plant *Crateva adansonii* affirm their anti-inflammatory potential screened through in vitro and in silico approaches [14, 15]. Main objectives of the present study is to examines acute toxicity evaluation of *Crateva adansonii* leaf extracts and in vivo anti-inflammatory and analgesic potential of lupeol isolated from Indian traditional medicinal plant *Crateva adansonii* through kit assay quantification method for inflammatory marker enzyme myeloperoxidase, inflammatory product PGE_2_, and eight different cytokines associated with inflammation determined in serum samples of experimental animals.

## Methods

### Plant extracts and lupeol fraction preparation

Shade-dried healthy leaves (initial quantity 400 g) of *Crateva adansonii* was pulverized into powder. Then the powder is used to prepare methanolic and chloroform leaf extracts of *Crateva adansonii* (ME, CE) using the soxhlet apparatus independently. Lupeol fraction (LF) (6 g with 15%yield) containing marker compound of the plant lupeol is isolated from 40 g of CE using a silica gel glass column chromatography procedure (70–230 mesh size, 48 × 2 cm). Column elution was performed with n hexane, ethyl acetate, and methanol solvents and the obtained fractions were subjected to TLC and confirm the existence of lupeol compound in the fraction and utilized for the study [15]. Three samples from *Crateva adansonii* ME, CE, and LF were used for further analysis.

### Animals and ethical clearance

One hundred eighty-nine numbers of Wistar albino rats were purchased from CPCSEA registered breeder Kerala Verterinary University, Mannuthi, Kerala, India and utilized for the study. All experimental rats were 8 weeks old and weighs 180–250 g maintained at standard conditions of 30–70% humidity and 26 ± 1 °C temperature in 12 h alternative dark/light cycles. All animals were allowed to feed with standard pelleted rat chaw. All the experimental protocols were done according to the Ethics Committee of Nandha College of Pharmacy, Erode, Tamilnadu, India (688/PO/Re/S/02/CPCSEA).

### Acute toxicity study

Acute toxicity of *C*. *adansonii* leaf extracts was determined as per the OECD guideline test no 423 [16]. Wister albino rat was utilized for acute toxicity study. Animals administered with different doses (100–2000 mg/kg) of *Crateva adansonii* leaf extracts (ME and CE) using 0.1% CMC as vehicle solution through peroral route were monitored for its behavioral and mortality rate, which will help to find out the lethality dose of the plant leaf extracts. Additional healthy control rat group was maintained without administering plant leaf extracts. For each leaf extract dose, three rats were employed then monitored for 3 days and mortality rate in two of three rats considered for acute toxicity evaluation whereas behavioral changes monitored and considered in all tested rats.

### Evaluation of in vivo anti-inflammatory activity

#### Animal grouping and carrageenan-induced rat paw edema model

Wistar albino rats were randomly split into seven groups from group I to group VII; each group has six animals (*n* = 6). Group I animals received vehicle (0.1% CMC) through the intragastric tube (control), group II animals receive indomethacin 20 mg/kg (reference standard), whereas groups III–VI animals receive 200, 400 mg/kg of ME and CE of *C*. *adansonii* respectively, and group VII animals receive LF 100 mg/kg (per oral route). Acute anti-inflammatory potential of *Crateva adansonii* was determined by the standard carrageenan rat paw edema model [17]. Animals in all groups were administered and challenged with 1% carrageenan (solubilized in saline solution and injected into sub-planter region of rat). Experimental animals paw thickness was measured from 0 min to 3 h at every half an hour interval using a plethysmometer. Inhibition of rat paw thickness was measured and expressed in percentage for all experimental animals were recorded. The percentage of inhibition calculated by the following formula; % Inhibition = 100 (1- Test mean paw thickness / control mean paw thickness).

#### Cotton pellet-induced granuloma model

Chronic anti-inflammatory activity of *Crateva adansonii* was determined using cotton pellet-induced granuloma model as per the standard method [18]. Briefly, sterile autoclaved two cotton pellets weighing 7 mg implanted to anesthetized Wister albino rat in the dorsal region of each axilla. Totally, seven experimental groups and six rats were employed for each experimental group. Animal grouping and leaf sample, standard drug dosage is similar as that of carrageenan-induced rat paw model (group I control; group II indomethacin 20 mg/kg; groups III–VI ME and CE 200 and 400 mg/kg; group VII LF 100 mg/kg). Further, 0.1% CMC was utilized as vehicle solution administered through peroral route. All experimental animals were maintained in standard conditions up to 7 days after cotton pellet implantation. On the 8^th^ day, all animals were sacrificed using excess thiopental sodium to collect granulomatous tissues formed around the cotton pellet. The wet and dry weight of granulomatous tissues was recorded for each experimental animal group. Result values are statistically analyzed by Dunnett’s “*t*” test using GraphPad software and the values were considered significant at *p* < 0.05.

#### Determination of PGE_2_ and cytokine marker levels in rat paw edema exudates

Prostaglandin E_2_ (PGE_2_) level in carrageenan-challenged paw edema exudates were determined using the PGE_2_ ELISA kit assay. The experimental animals from the entire group were sacrificed using excess thiopental sodium after 3 h; carrageenan challenge and hind paws were excised and inflammatory exudates were collected from centrifugation of excised hind paw [19]. In brief, a substrate solution was added to the assay wells and incubates for color development and the reaction is stopped to measure color intensity at 450 nm. All the reagents and sample preparation was done as per the kit assay procedure [20].

Eight different inflammatory cytokine marker levels of rat paw edema exudates were determined using signosis Rat Inflammation ELISA strip assay (catalog no: EA-4002). In brief, control or test samples were added and incubated to each well coated with primary antibody against cytokine then washed with assay buffer. Add biotin-labeled antibody to each well followed by streptavidin-HRP conjugate and substrate solution to incubate with gentle shaking for color development. The reaction was terminated by adding the stop solution. The density of the color developed was read at 450 nm using a microplate reader within 30 min of the assay. PGE_2_ level were expressed as pg/ml of rat paw exudates whereas all other cytokine levels were expressed in relation to optical density at 450 nm of rat paw exudates.

#### Myeloperoxidase enzyme assay

Inflammatory marker enzyme MPO was assayed in rat paw edema tissues using the standard method with slight modification [21]. Rat paw tissues was homogenized using 10% phosphate buffer then centrifuged and collect supernatant used for MPO assay. In brief, a known aliquot of the sample was mixed with phosphate buffer containing *O*-dianisidinedihydrochloride and hydrogen peroxide. The sample absorbance was measured spectrophotometrically at 460 nm and their triplicate results were recorded. MPO level were expressed as U/mg of rat paw tissue protein.

### Evaluation of in vivo analgesic activity

#### Hot plate assay

Hot plate assay is used to evaluate the analgesic activity of *Crateva adansonii* plant leaf extracts and its lupeol fraction was done as per the previously reported standard method [22]. Totally, seven experimental groups and six rats were employed for each experimental group. Animal grouping and leaf sample dosage is similar as that of carrageenan induced rat paw model with exception of 10 mg/kg of pentazocine employed as a standard drug (group I control; group II pentazocine 10 mg/kg; group III–VI ME and CE 200 and 400 mg/kg; group VII LF 100 mg/kg) administered through peroral route. In brief hot plate, temperature was maintained at 55 ± 0.5 °C. The time taken for a mouse to shake, lick, or jumping of the hind paw is considered as pain indicators. The pre-treatment reaction time of each mouse was recorded initially before sample administration. Animal groups were administered orally for six consecutive days as per the dosage form as in anti-inflammatory assay. Post-treatment reaction time for each animal is recorded at 30, 60, 90,120 min on 7^th^ day after oral administration. The percentage of inhibition is calculated by the difference between post-treatment and pre-treat time divided by pre-treatment time.

#### Acetic acid-induced writhing assay

Acetic acid-induced writhing assay is used to evaluate analgesic activity of *Crateva adansonii*. Totally, seven experimental groups and six rats were employed for each experimental group. Animal grouping and leaf sample, standard drug dosage is similar as that of carrageenan-induced rat paw model (group I control; group II pentazocine 10 mg/kg; group III–VI ME and CE 200 and 400 mg/kg; group VII LF 100 mg/kg) administered through peroral route. In brief, each experimental animal mouse was administered with 1%acetic acid (1 ml/100 g) intraperitoneally. The mice were observed for writhes and the total number of writhes in control and treated group animal for 20 min after the last injection is recorded. Percentage of analgesic activity is calculated by the difference between writhing count of control and treated groups divided by writhes in control groups. Statistical data were analyzed by using one-way ANOVA followed by Dunnett’s “*t*” test using the software Graph Pad and results values were considered significant (*p* < 0.05).

#### Molecular docking study

Autodock (Version 4) was employed for the present study and the calculations were carried out by Autodock tools [23]. Receptor-ligand obtained are ranked using an energy-based scoring functions. 3D crystal structures of nicotinic acetylcholine receptor nAChR (PDB 2KSR) as analgesic target and COX-2 enzyme receptor (PDB 6COX) as inflammatory molecular targets was retrieved from Protein data Base (PDB www.rcsb.org). Lupeol and reference drug for analgesic and inflammatory protein targets such as pentazocine and indomethacin 2D structures were retrieved from PubChem database (https://pubchem.ncbi.nlm.nih.gov/) [24]. Retrieved 2D SDF file formats were submitted to “Online SMILES convertor and Structure file generator” and converted into standard 3D PDB formats for further in silico analysis [25]. The grid map that represents the protein binding sites for docking was calculated using AutoGrid. The grid size of 98 × 74 × 76 points in each dimension was set for COX-2; the grid size for nAChR was set to 31 × 60 × 62 points in each dimension covers entire target protein. The grid points space (0.375 Å) was set by using AutoGrid and Gasteiger charges were measured by AutoDock tools. Assessment of docking (~ 100 times), size of population (150), energy evaluation (maximum number 250,000) generations (maximum number 27,000), rate of mutations (0.02), rate of cross-over (0.8), value of elitism (1), and other parameters as default values were established using the autotors utility of the AutoDock tool. The results of molecular docking studies and their molecular protein target-drug interactions were visualized and analyzed using receptor-ligand interaction options in Discovery Studio v2.5 software.

## Result

### Acute toxicity study

Acute toxicity study was conducted to determine the safe dosage of pharmacologically active principles from plant extracts to animal and human subjects. In the present study, Wister albino rats were employed and administered with different doses of two different leaf extracts (ME and CE) of *Crateva adansonii* to test their lethality. There are no marked behavior changes and zero mortality rate in all tested male Wister albino rat administered with ME and CE leaf extracts up to 2000 mg/kg body weight. Results proved that the administration of ME and CE from *Crateva adansonii* up to the 2 g/kg dose were safe to be used for our various anti-inflammatory investigations through in vivo model, in a single administration. So we have chosen two optimal concentrations (200 and 400 mg/kg) for both ME and CE plant leaf extracts, whereas dosage of 100 mg/kg LF is used for further anti-inflammatory and analgesic study.

### Evaluation of in vivo anti-inflammatory activity

#### Carrageenan-induced rat paw edema model

**Fig. 1:**
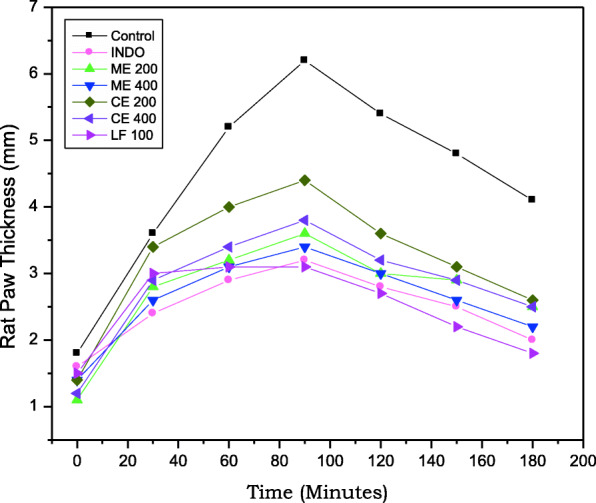
Rat paw thickness (mm) of experimental animal groups at different time intervals assessed through acute anti-inflammatory activity

**Fig. 2:**
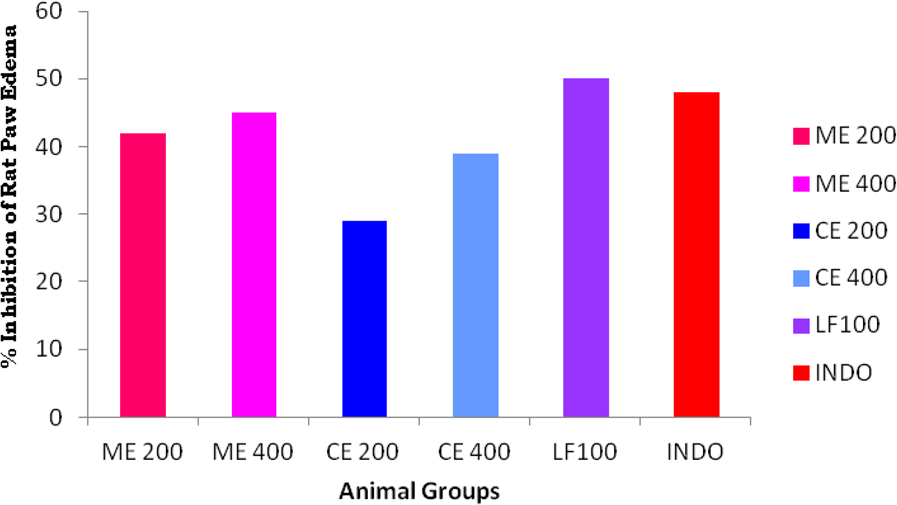
Percentage of Inflammation inhibition by *Crateva adansonii* leaf extracts, lupeol fractions (ME, CE, and LF), and reference standard in Caragennan induced rat paw edema model

The results of paw thickness differences in mm obtained from Carrageenan-induced rat paw edema in vivo model were depicted in Table 1 and Fig. 1. The maximal percentage of rat paw inhibition (50%) was recorded in group VII animals receiving LF than group II animals receiving reference standard indomethacin shows 48% of inhibition in rat paw edema. Experimental animals from groups III and IV receiving methanolic leaf extract of the plant shows 42% and 45% of rat paw edema inhibition at 3 h respectively. Whereas animals from groups V and VI receiving chloroform leaf extracts of *C*. *adansonii* shows 29% and 39% of rat paw edema inhibition respectively at 3 h (Fig. 2).

**Table 1:** Acute anti-inflammatory activity of *Crateva adansonii* leaf extracts and its Lupeol containing LF in male Wistar albino rat

Groups	Drug Treatment	Paw thickness in mm							% Inhibition at 3 h
		0 h	½ h	1 h	1½ h	2 h	2½ h	3 h	
I	Control 0.1% CMC	1.8 ± 0.02	3.6 ± 0.02	5.2 ± 0.04	6.2 ± 0.02	5.4 ± 0.02	4.8 ± 0.02	4.1 ± 0.02	-
II	Indomethacin 20 mg/kg	1.6 ± 0.04	2.4 ± 0.04**	2.9 ± 0.01**	3.2 ± 0.01**	2.8 ± 0.01**	2.5 ± 0.04**	2.0 ± 0.01*	48
III	ME 200 mg/kg	1.1 ± 0.02	2.8 ± 0.04**	3.2 ± 0.04**	3.6 ± 0.04**	3.3 ± 0.02**	2.9 ± 0.02*	2.5 ± 0.01**	42
IV	ME 400 mg/kg	1.4 ± 0.04	2.6 ± 0.01**	3.1 ± 0.01**	3.4 ± 0.03**	3.0 ± 0.01**	2.6 ± 0.01**	2.2 ± 0.01**	45
V	CE 200 mg/kg	1.4 ± 0.02	3.4 ± 0.01*	4.0 ± 0.02**	4.4 ± 0.04**	3.6 ± 0.02**	3.1 ± 0.02**	2.6 ± 0.03**	29
VI	CE 400 mg/kg	1.2 ± 0.01	2.9 ± 0.02**	3.4 ± 0.02*	3.8 ± 0.02**	3.2 ± 0.04**	2.9 ± 0.04**	2.5 ± 0.02*	39
VII	LF 100 mg/kg	1.5 ± 0.03	3.0 ± 0.03**	3.1 ± 0.01**	3.1 ± 0.01**	2.7 ± 0.01**	2.2 ± 0.02**	1.8 ± 0.03**	50

The data are presented as mean ± SEM, *n* = 6, *p < 0.05, **p < 0.01 vs. control. Data were analyzed by using one-way ANOVA followed by Dunnett’s “*t*” test using the software Graph Pad

*ME* Methanolic extract of *C*. *adansonii* leaf, *CE* Chloroform extract of *C*. *adansonii* leaf, *LF* Lupeol fraction

#### Cotton pellet induced granuloma model

**Fig. 3:**
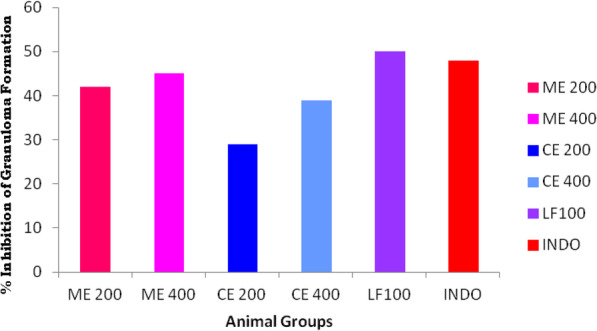
Percentage inhibition of granuloma formation by *Crateva adansonii* leaf extracts, lupeol fractions (ME, CE, and LF), and reference standard in chronic anti-inflammatory study

Chronic anti-inflammatory potential of *Crateva adansonii* was analyzed by cotton pellet-induced granuloma in vivo model and their results were presented in Table 2 and Fig. 3. Wet, dry, and transudative weights are measured for each experimental animal groups. Highest wet, dry, and transudative weights (214.66 mg, 58.62 mg, and 156.04 mg respectively) were monitored in group I control animal receiving 0.5% CMC as a vehicle control. The highest percentage of inhibition (33.96%) was measured in group VII animals receiving LF than all other tested animal groups ranging from 10.84 to 25.18% of granuloma inhibition.

**Table 2:** Chronic anti-inflammatory activity of *Crateva adansonii* leaf extracts and its Lupeol containing LF in male Wister albino rat

Groups	Drug treatment	Wet qWEIGHT (mg)	Dry weight (mg)	Transudative weight (mg)	Percentage inhibition
I	Control 0.1%CMC	214.66 ± 8.36	58.62±1.69	156.04	–
II	Indomethacin (20 mg/kg)	159.44 ± 5.32*	42.69 ± 2.27*	116.75**	25.18
III	ME 200 mg/kg	186.67 ± 7.38	47.55 ± 1.97	139.12	10.84
IV	ME 400 mg/kg	167.26 ± 6.34*	41.33 ± 2.26*	125.93*	19.29
V	CE 200 mg/kg	180.62 ± 3.77	46.30 ± 3.15	134.32	13.92
VI	CE 400 mg/kg	162.47 ± 6.37*	41.66 ± 1.96*	120.81*	22.58
VII	LF 100 mg/kg	155.36 ± 5.75*	52.31 ± 2.55*	103.05**	33.96

The data are presented as mean ± SEM *n* = 6. *p < 0.05, **p < 0.01 vs. control. Data were analyzed by using one-way ANOVA followed by Dunnett’s “*t*” test using the software Graph Pad

*ME* Methanolic extract of *C*. *adansonii* leaf, *CE* Chloroform extract of *C*. *adansonii* leaf, *LF* Lupeol fraction

#### Determination of PGE_2_ level and cytokine marker levels

**Fig. 4:**
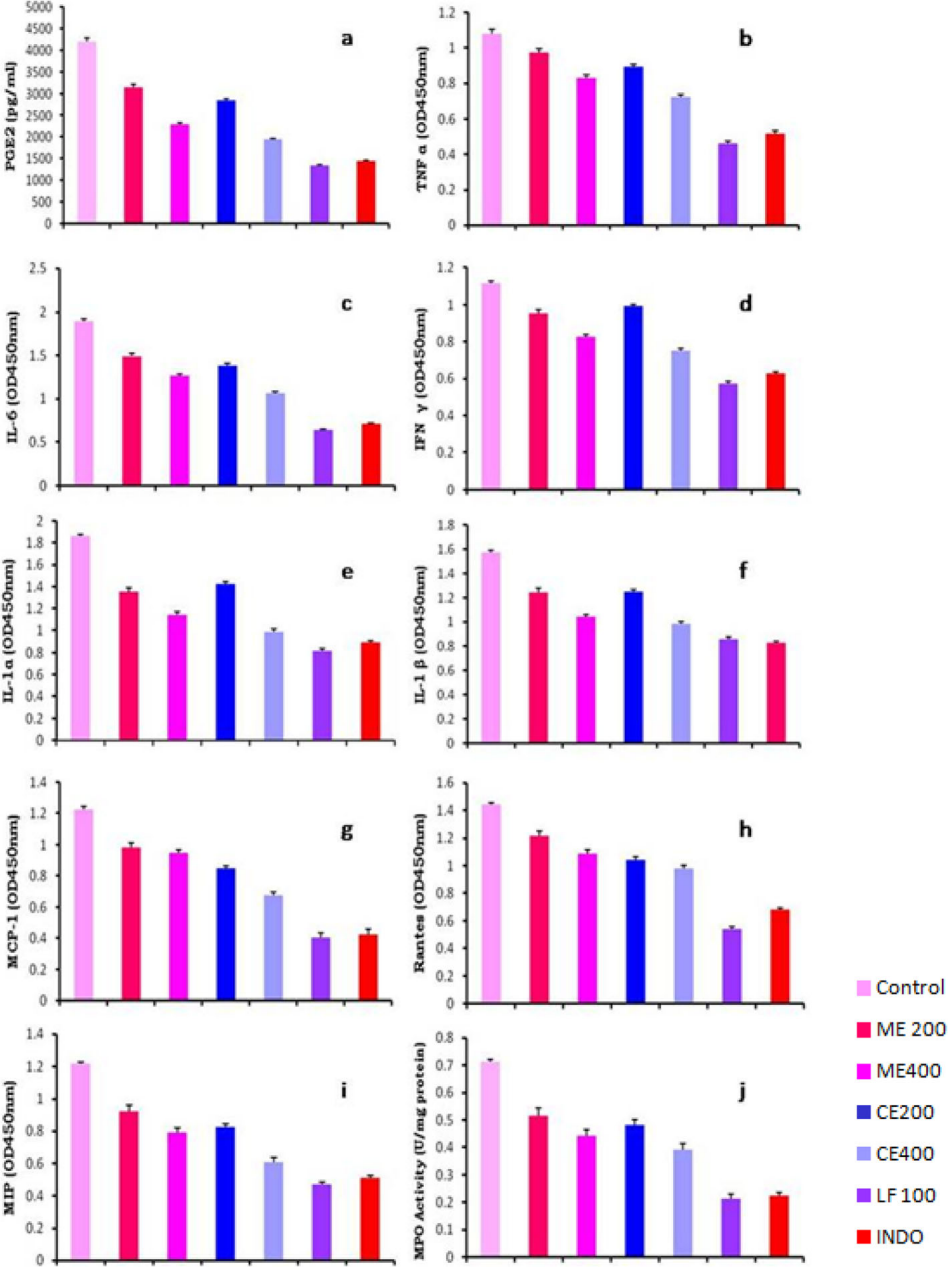
**a**-**j** Inflammatory enzymes (PGE2 and MPO) and cytokines levels of Carrageenan challenged animals

The level of prostaglandin E_2_ was measured in exudates collected from experimental animal paws and their results were presented in Fig. 4a. “Prostaglandin E_2_ level in control animals is increased four-fold” when compared with the experimental animal groups receiving lupeol fractions (LF). Group II to VII experimental animal receiving *Crateva adansonii* plant leaf extracts and its lupeol fractions shows the significant dose-dependent reduction of PGE_2_ level 35, 75, 55, 68, 46, and 32% of inhibition respectively. These result findings served as a benchmark to prove the anti-inflammatory potential of leaf extracts and its fraction (ME, CE, and LF) for efficient blocking of the prostaglandin inflammatory pathway.

Eight cytokine inflammatory markers such as interferon-gamma (IFNγ), interleukin-1alpha, beta (IL-1α and 1β), interleukin-6 (IL-6), tumor necrosis factor alpha (TNF α), macrophage inflammatory protein (MIP), monocyte chemoattractant protein-1 (MCP-1), and regulated upon activation normal T cell expressed and presumably secreted (RANTES) were analyzed by Rat Signosis ELISA assay, and the results were presented in Fig. 4b–i. Marked reduction of eight inflammatory cytokines levels of test group’s animal receiving ME, CE, and LF than the animals receiving reference indomethacin. LF causes nearly 2- to 4-fold fall in all inflammatory cytokine absorbance level at 450 nm when compared with all other tested samples and reference indomethacin. Among all tested groups, experimental animals receiving a dose of 100 mg/kg LF show a potential reduction in cytokine levels except IL-1β.

#### Assessment of myeloperoxidase activity

Inflammatory marker enzyme myeloperoxidase (MPO) level was assessed in carrageenan-challenged experimental animals and their results were depicted in Fig. 4j. Group VII experimental animals receiving lupeol fractions resulted in 4-fold of MPO enzyme activity inhibition when compared with the control group animal enzyme activity. Animals receiving LF show significant reduction in MPO enzyme production when compared with all other tested group animals including reference indomethacin.

### Evaluation of in vivo analgesic activity

#### Hot plate assay

**Fig. 5:**
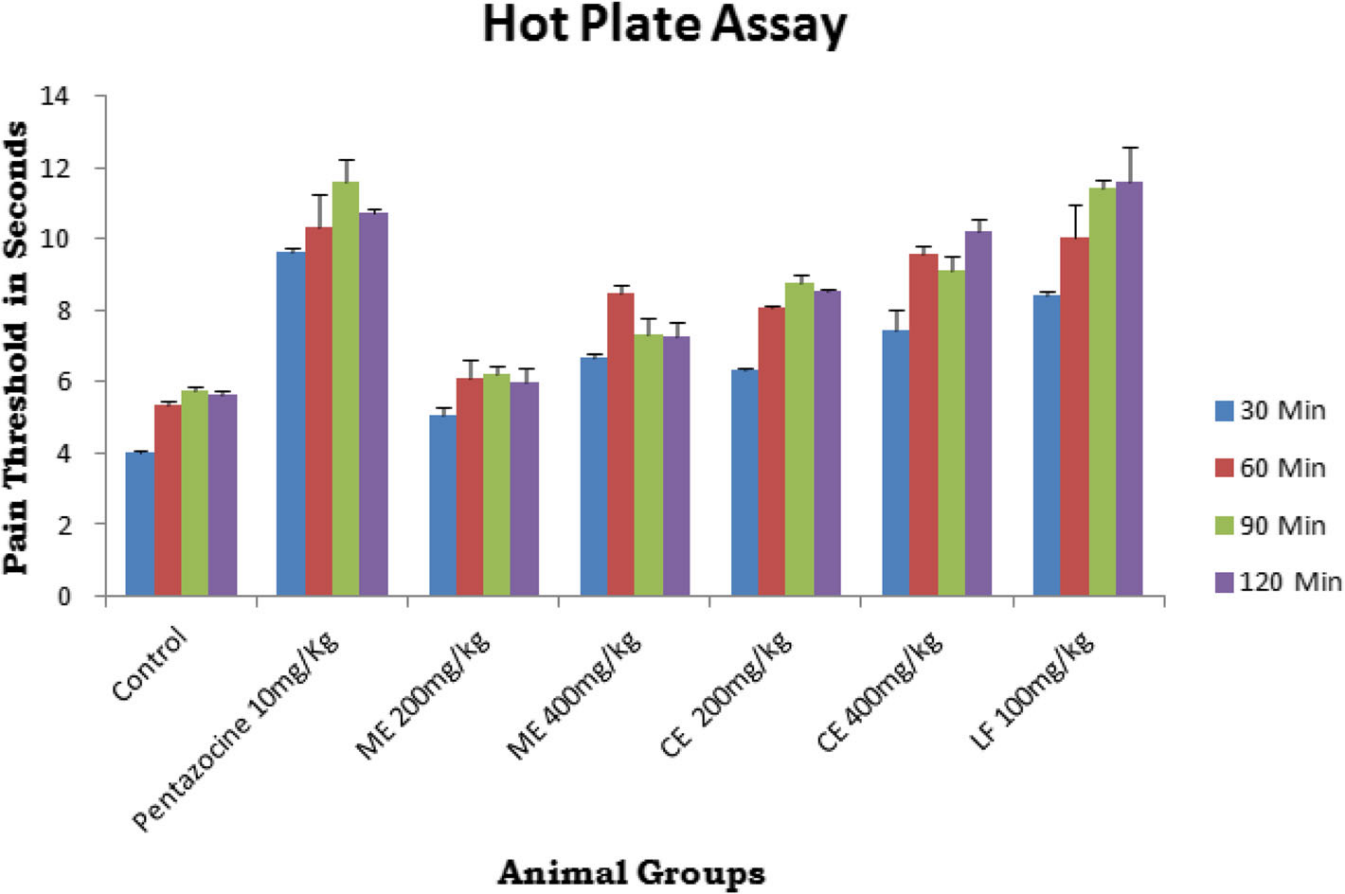
Effect of *Crateva adansonii* leaf extracts and its Lupeol containing LF on hot plate assay in mice

The results of hot plate assay of experimental animals receiving *Crateva adansonii* leaf extracts and its LF fractions were displayed in Table 3 and Fig. 5. Animals receiving LF shows a significant (*p* < 0.001) analgesic effect when compared with all other tested groups. LF-treated animal shown remarkable pain threshold values at each time interval after 30, 60, 90, and 120 min (8.40, 10.06, 11.45, and 11.60 s respectively) which is very closer to the pain threshold times of narcotic pentazocine standard (9.66, 10.35, 11.63, and 10.76 s respectively), whereas the chloroform leaf extract receiving animals shows considerable analgesic activity compared to the animals receiving methanolic leaf extracts. Further study reveals that LF from *Crateva adansonii* reveals equipotent pain inhibition at 30, 60, and 90 min with standard, but at the end 120 min, LF shows highest pain threshold time 11.60 ± 0.96 s and 10.76 ± 0.06 s respectively than standard pentazocine.

**Table 3:** Effect of *Crateva adansonii* leaf extracts and its Lupeol containing LF on hot plate assay in mice

Groups	Drug treatment	Pain threshold (in s)			
		30 min	60 min	90 min	120 min
I	Control 0.1%CMC	4.02 ± 0.02	5.33 ± 0.14	5.77 ± 0.09	5.65 ± 0.12
II	Pentazocine 10 mg/kg	9.66 ± 0.11**	10.35 ± 0.92***	11.63 ± 0.60***	10.76 ± 0.06***
III	ME 200 mg/kg	5.08 ± 0.22	6.09 ± 0.55	6.23 ± 0.24	5.96 ± 0.40
IV	ME 400 mg/kg	6.66 ± 0.12	8.47 ± 0.22*	7.30 ± 0.45*	7.28 ± 0.40*
V	CE 200 mg/kg	6.31 ± 0.09	8.06 ± 0.07*	8.76 ± 0.22*	8.54 ± 0.05*
VI	CE 400 mg/kg	7.45 ± 0.54*	9.60 ± 0.22**	9.10 ± 0.43**	10.22 ± 0.32***
VII	LF 100mg/kg	8.40 ± 0.15**	10.06 ± 0.88***	11.45 ± 0.22***	11.60 ± 0.96***

The data are presented as mean ± SEM *n* = 6. *p < 0.05, **p < 0.01, and ***p < 0.001 vs. control. Data were analyzed by using one-way ANOVA followed by Dunnett’s “*t*” test using the software Graph Pad

*ME* Methanolic extract of *C*. *adansonii* leaf, *CE* Chloroform extract of *C*. *adansonii* leaf, *LF* Lupeol fraction

#### Acetic acid-induced writhing assay

Acetic acid-induced writhing assay results were shown in Table 4. It reveals that LF possesses equipotent analgesic activity (69.05%) when compared with reference standard indomethacin (70.05%). Highly significant (*p* < 0.001) analgesic activity with less writhing count was seen in the standard group (19.60 ± 0.95) followed by the LF group (20.26 ± 0.98) and group VI experimental (25.35 ± 1.55) animals.

**Table 4:** Effect of *Crateva adansonii* leaf extracts and its Lupeol containing LF on acetic acid-induced writhing in mice

Groups	Drug treatment	Writhing count for 20 min	Percentage of analgesic activity
I	Control 0.1%CMC	65.45 ± 2.50	–
II	Indomethacin 20 mg/kg	19.60 ± 0.95***	70.05%
III	ME 200 mg/kg	48.33 ± 2.22*	26.16%
IV	ME 400 mg/kg	38.671.75**	40.92%
V	CE 200 mg/kg	36.76 ± 2.22**	43.83%
VI	CE 400 mg/kg	25.35 ± 1.55***	61.26%
VII	LF 100 mg/kg	20.26 ± 0.98***	69.05%

The data are presented as mean ± SEM *n* = 6. *p < 0.05, **p < 0.01, and ***p < 0.001 vs. control. Data were analyzed by using one-way ANOVA followed by Dunnett’s “*t*” test using the software Graph Pad

*ME* Methanolic extract of *C*. *adansonii* leaf, *CE* Chloroform extract of *C*. *adansonii* leaf, *LF* Lupeol fraction

#### Molecular docking analysis

**Fig. 6:**
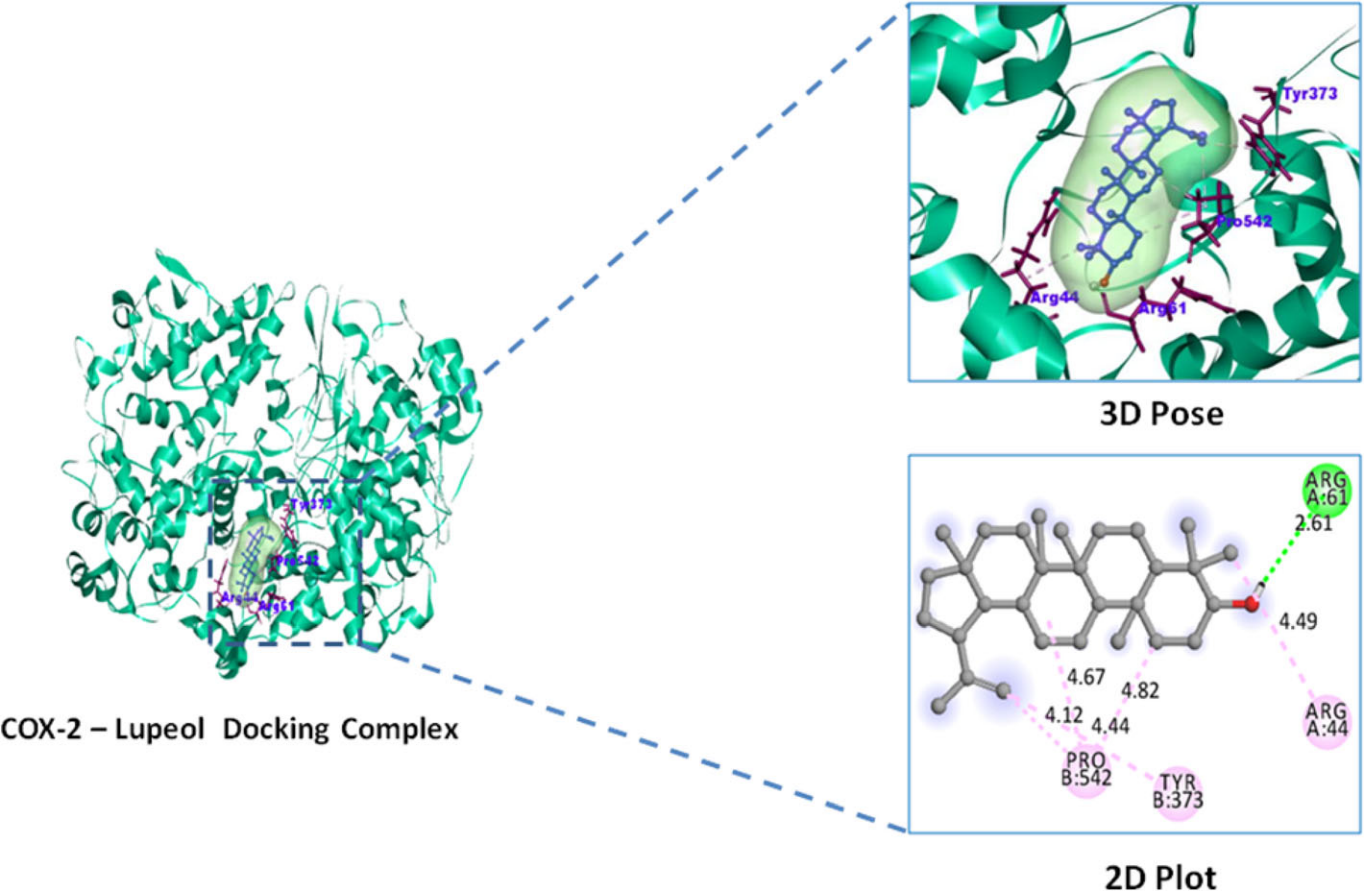
Inflammatory receptor COX-2–Lupeol docking complex 3D pose and their 2D interaction plot

**Fig. 7:**
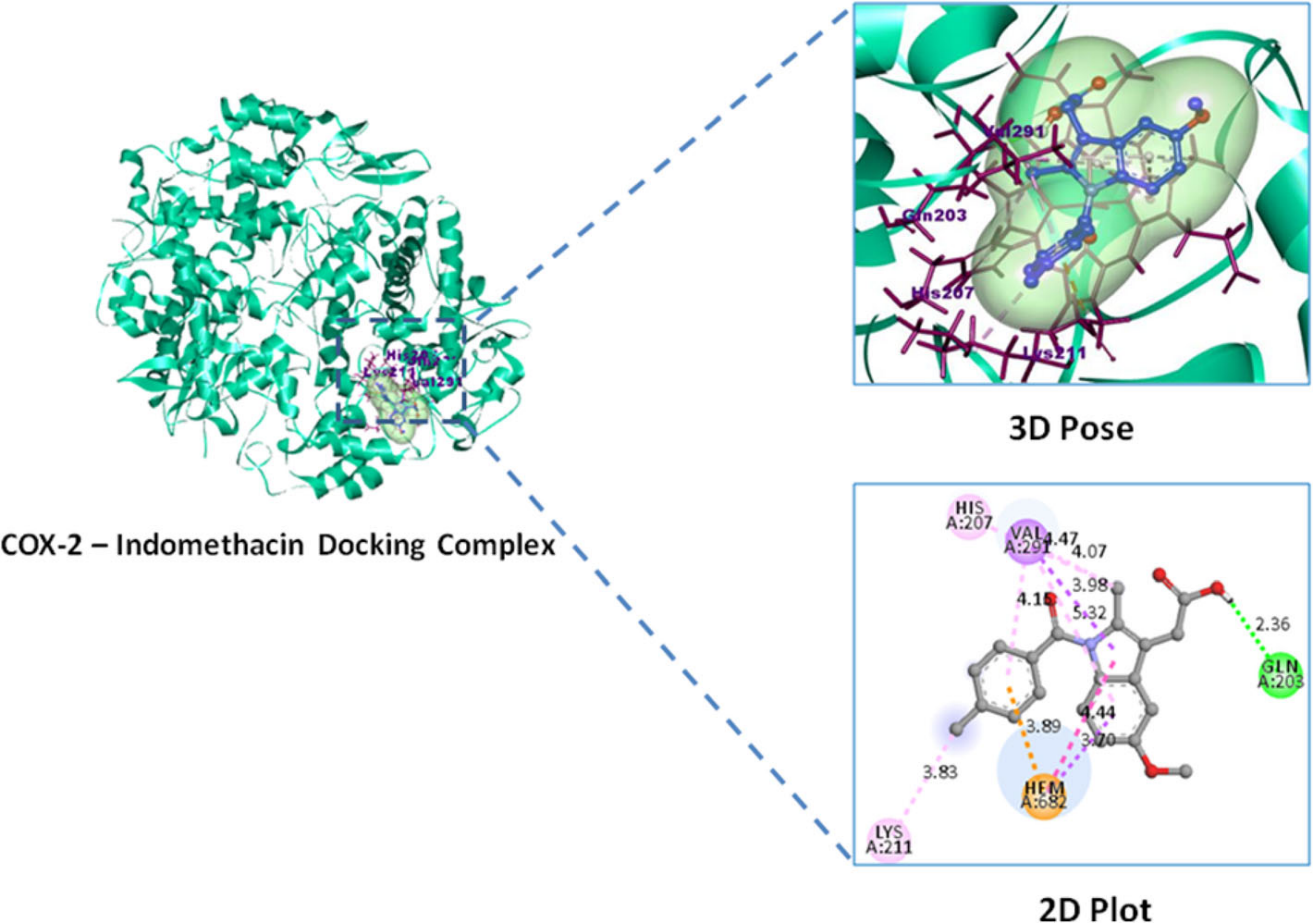
Inflammatory receptor COX-2–Indomethacin (reference standard) docking complex 3D pose and their 2D interaction plot

**Fig. 8:**
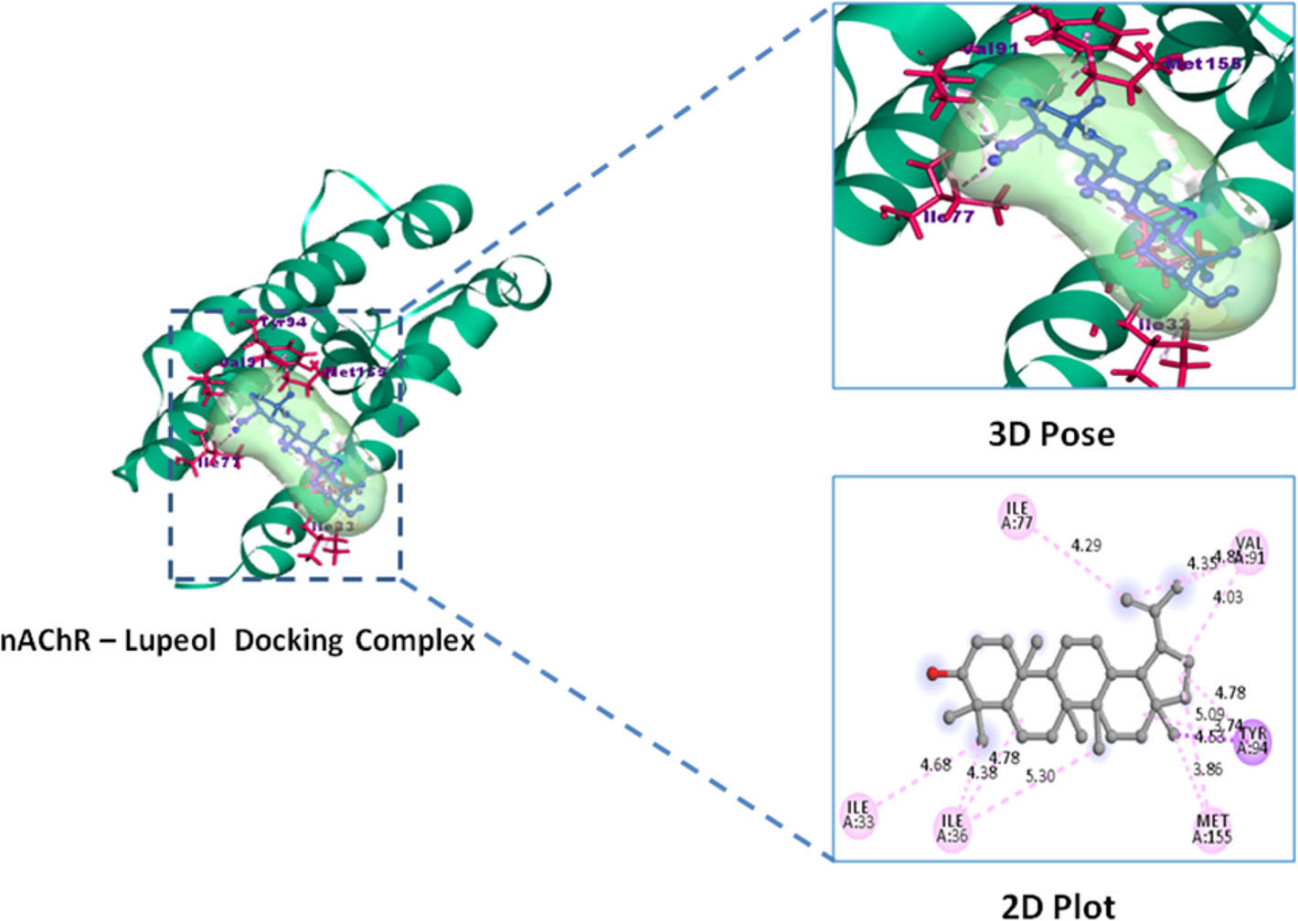
Analgesic receptor nAChR–Lupeol docking complex 3D pose and their 2D interaction plot

**Fig. 9:**
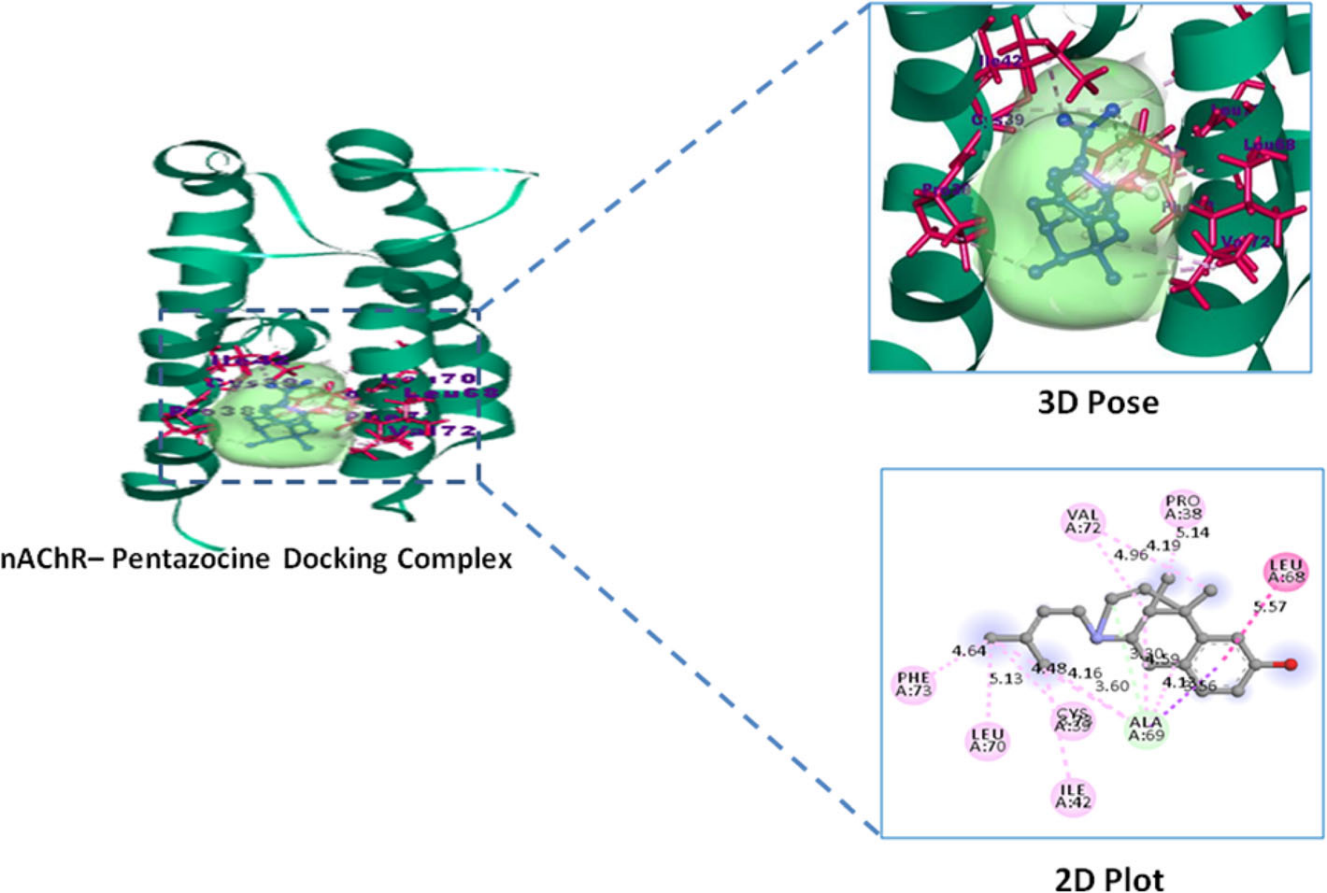
Analgesic receptor nAChR–Pentazocine (reference standard) docking complex 3D pose and their 2D interaction plot

Lupeol exhibited highest binding affinity and docking score − 9.0 Kcal/mol against inflammatory COX-2 and − 8.5 Kcal/mol against analgesic (nAChR) protein targets over their reference standard drugs pentazocine − 7.0 Kcal/mol and indomethacin − 8.4 Kcal/mol with nAChR and COX-2 receptors respectively (Table 5). High binding affinity of lupeol with inflammatory marker enzyme Cycloxygenase-2 (COX-2) is favored by the alkyl and hydrogen bonded interactions with COX-2 residues of Arg44, Tyr373, Pro542, and Arg61 respectively. Similarly lupeol form alkyl interactions with Ile33, Ile36, Ile77, Val91, Tyr94 residues of analgesic nicotinic acetyl choline receptor. Whereas reference standard indomethacin possess one hydrogen bonded interactions Gln203 and three alkyl bonded His207, Lys211, Val291 interactions with COX-2 enzyme, similarly pentazocine possess alkyl interactions with Pro38, Cys39, Ile42, Leu68, Ala69, Leu70, Val72, Phe73 residues of analgesic receptor nAChR (Figs. 6, 7, 8, and 9).

**Table 5:** Binding affinity and molecular interactions of Lupeol and reference standard drug against analgesic and inflammatory target proteins

Docking complex	Binding affinity Kcal/mol	Residues involved in interaction
COX-2–Lupeol	− 9.0	Arg44,Arg61*,Tyr373,Pro542
COX-2–Indomethacin	− 8.4	Gln203*,His207,Lys211,Val291
nAChR–Lupeol	− 8.5	Ile33,Ile36,Ile77,Val91,Tyr94,Met155
nAChR–Pentazocine	− 7.0	Pro38,Cys39,Ile42,Leu68,Ala69,Leu70,Val72,Phe73

Residues with * symbol indicates the hydrogen bonded interactions

Residues without any symbol indicates Alkyl bonded interactions

## Discussion

In our present study, we seek the pharmacological evidence for analgesic and anti-inflammatory potential of lupeol isolated from *Crateva adansonii* leaf extract which was proven through different in vivo model study in order to promote the cheapest source of key anti-inflammatory molecule from natural origin and alternate the existing NSAIDs drug usage for chronic inflammatory diseases. Natural products from medicinal plants are likely to be safe without any compromising health effect, and thus widely used as self-medication [26]. However, the safety and toxicity assessment of natural products is done through OECD guidelines to provide scientific evidence for human consumption, which is being carried out in various experimental animal models to evaluate their safety for future human use [27].

After carrageenan stimulation, paw thickness of experimental animals is increased gradually up to 0–3 h which is due to the biphasic inflammatory mechanism which leads to the activation and accumulation of inflammatory mediators. Initial (within 6 h) and final (after 6 h) phase causes the inducible nitric oxide synthase (iNOS) and COX-2 enzyme elevation respectively. Carrageenan-induced rat paw edema method is a widely employed method to investigate preliminary screening method to assess and measure the anti-inflammatory effect of natural products [28, 29]. Carrageenan is a sulfated polysaccharide substance used to produce inflammatory immune response in experimental animals leading to the release of pro-inflammatory mediators such as prostaglandin, histamine, bradykinin, leukotrienes, etc. [30]. The mechanism of carrageenan-induced inflammation in two-phase response, serotonin, and histamine released during the first phase followed by the release of prostaglandins, lysosome enzymes during the second phase [31–33]. Chloroform leaf extract fraction (LF) of *C*. *adansonii* shown maximal percentage (50%) of inhibition of rat paw thickness at 3 h and it efficiently blocks later phase pro-inflammatory cytokines release in acute inflammation model. The LF possess marker phytocompound of the plant; lupeol act as clinically active anti-inflammatory agent block pro-inflammatory cytokines and enzymes [34].

Cotton pellet-induced granuloma model is a routinely employed standard method to assess chronic anti-inflammation activity of natural compounds; dry and wet weight of cotton pellet is used to measure the granulomatous tissue formation. Administration of *Crateva adansonii* leaf extracts and leaf extract fraction has been observed to inhibit the granulomatous tissue formation; ultimately, it reduces the weight of wet cotton pellets in a dose-dependent manner remarkably the LF produced more inhibitory effect in granulomatous tissue formation than the reference standard. The amount of granulomatous tissue formed is directionally proportional to the rate of accumulation of inflammatory products such as macrophages, neutrophils, fibroblasts, etc. [35, 36]. The degree of the anti-inflammatory effect of the sample is directly proportional to the percentage of inhibition of granulomatous tissue formation. Group VII animals (33.96% inhibition) receiving LF appears to be more potent than group II animals (25.18%) receiving reference standards in inhibiting the dry weight of cotton pellets. Triterpenes found to stabilize mast cell membranes and inhibit the infiltration of the number of inflammatory cells at the site of inflammation [37]. Chloroform leaf extract fraction (LF) of *Crateva adansonii* possess marker triterpene compound namely lupeol may be responsible for the marked anti-proliferative effect on granulomatous tissue formation. *Crateva adansonii* plant marker compound lupeol was found to inhibit transcriptional factor NF-κB and other molecular targets of inflammation [38].

LF significantly offset the carrageenan-induced PGE_2_ production in paw edema exudates of group VII animals. Prostaglandin E_2_ is a major inflammatory mediator involved in most of the inflammatory disease conditions, so the inhibition of PGE_2_ is considered as the best way to treat most of the diseases associated with inflammation [39]. Prevention of neutrophils infiltration at inflammation site is monitored through myeloperoxidase enzyme level in experimental animals serum of rat paw edema model revealed that lupeol fraction from *Crateva adansonii* evoke excellent MPO inhibition than reference standard animal group. Taken together, inhibition of inflammatory enzyme (PGE_2_ and MPO) and pro-inflammatory cytokines by *Crateva adansonii* leaf extracts and lupeol fractions could negatively affect monocyte and leukocyte migration, which is scientifically evidenced by the hidden molecular mechanisms of the anti-inflammatory effect of the plant *Crateva adansonii*.

Group VII experimental animals receiving LF shows a significant reduction of cytokine levels, which once again proves the potential of LF in blocking inflammatory cytokine production cascade over the reference standard. Cytokines act as a biomarker for different inflammatory disease conditions and their cut off values will help to diagnose particular inflammatory disease conditions [40].

Protective effect of lupeol fractions against the carrageenan-induced rat paw edema is observed through screening of various inflammatory markers in the study. Lupeol fraction of *Crateva adansonii* inhibit and suppress tumor necrosis factor-α (TNF-α) and IL-1β [41]. Both TNF-α and interleukin-1 (IL-1) play major role in pathogenesis of inflammatory disorders such as rheumatoid arthritis. These proinflammatory cytokines and interleukins are involved in osteoclast differentiation, inflammation, and bone erosion [42]. Reduction of these inflammatory mediators will prevent side effect and further clinical manifestation of arthritic inflammations. TNF-α is an important inflammatory cytokine marker activates the expression of other cytokines, chemokines, adhesion molecules, and neutrophil [43]. So the effect of LF from *Crateva adansonii* on TNF-α was investigated. It was shown that LF causes significant reduction of TNF-α, IL-1, and IL-6 cytokine levels which could inhibit the infiltration and activation of macrophage at inflammatory sites. Our study reveals that lupeol fractions of *Crateva adansonii* evoke promising inflammation inhibition and suggested its role in the suppression of inflammatory mediators.

The study was further extended to evaluate LF possible effects on monocyte and leukocyte migration at the inflammatory sites. This was done through assessing the effect of LF on monocyte chemoattractant protein-1 (MCP-1) and RANTES (regulated upon activation, normal T cell expressed and secreted). MCP-1 is a pro inflammatory cytokine responsible for the infiltration of monocytes at inflammatory sites [44]. Rise in RANTES production is closely associated with a broad range of inflammatory conditions. It acts by promoting leukocyte infiltration to the site of inflammation. RANTES induces activation of T cells [45] followed by amplified production of IL-2 and IFN-γ. In the current study, LF from *Crateva adansonii* alleviated carrageenan-induced RANTES production. As a result of RANTES inhibition by LF unable to activate T cell, this was observed through reduced IFN-γ production. Taken altogether, LF could remarkably affect and reduces monocyte and leukocyte migration as evident from alleviated MCP-1 and RANTES production. The level of macrophage inflammatory protein (MIP) in paw edema exudates of carrageenan-treated rats was found to be significantly reduced in LF pretreated rats.

Hot plate method is widely employed to screen and evaluate analgesic drug acting central nociceptive system [46, 47], which contribute significant pain generation upon inflammation and nerve injury. It is one of the models normally used for studying central nociceptive activity [48]. Analgesic potential of any anti-nociceptive agent acting on central nociceptive system causes a prolongation of hot plate latency period [49]. Lupeol fraction of the *Crateva adansonii* shows equipotent analgesic activity compared with opioid standard pentazocine acting on central nociceptive system. Narcotic analgesics inhibit both peripheral and central mechanism of pain, while NSAIDs inhibit only peripheral pain [50, 51]. Therefore, *Crateva adansonii* leaf extracts and lupeol fractions showed both types of pain inhibition. The analgesic effect of the plants in both models suggests that they have been acting through central and peripheral mechanism [48]. Groups VI and VII animals receiving chloroform leaf extract and LF of *Crateva adansonii* showed remarkable analgesic activity (10.22 ± 0.32 s and 11.60 ± 0.96 s at 120 min respectively) in hot plate assay; it indicates that LF and CE of plant *Crateva adansonii* possess excellent equipotent CNS analgesic efficiency and when compared with standard drug pentazocine (10.76 ± 0.06 s at 120 min).

Analgesic activity was assessed by acetic acid-induced writhing assay which is widely employed and reported valuable investigation method for assessing central and peripheral antinociceptive activity of pharmacological important substances and performed as a chemical pain model [52]. Prostaglandin level of peritoneal exudates of mice is elevated at 30 min after the injection of acetic acid [53, 54]. The degree of writhing obtained in the assay is well correlated with the sensitization of pain receptors followed by the elevated level of prostaglandin. Writhing assay results revealed that group VI animals receiving 400 mg CE (61.26%) and group VII animals receiving LF (69.05%) possess equipotent analgesic efficiency when compared with the standard drug pentazocine (70.05%).

Previously reported in silico studies of COX-2 with natural compounds (epiloganin, curcumin, lupeol) and celecoxib, indomethacin reveals that compounds from natural origin have anti-inflammatory therapeutic value that possess highest binding affinity with least docking scores than commercial reference standard drugs [55, 56]. Similarly, our present study has shown that lupeol phytocompounds isolated from *Crateva adansonii* leaf extract shows the higher binding affinity toward COX-2 enzyme than commercial available NSAIDs drugs including COX-2 selective inhibitors [57].

## Conclusion

The present study results well explains and support the folkloric usage of *Crateva adansonii* plant leaf in different inflammatory and nociceptive disease conditions in India. Acute toxicity screening reveals that no lethality was noticed to experimental rats by administration of *Crateva adansonii* leaf extract up to 2 g/kg. Further, the study demonstrates lupeol containing LF of the plant possess equipotent analgesic and anti-inflammatory activity compared with the standard drugs such as pentazocine and indomethacin. LF and leaf extract from *Crateva adansonii* shown to inhibit prostaglandin, inflammatory enzyme myeloperoxidase, and inflammatory cytokines release associated with pain and inflammatory pathways. Finally, LF from *Crateva adansonii* has proven its remarkable analgesic effect in both hot plate and acetic acid-induced writhing mice in vivo models. The present study provides in vivo pharmacological evidences for analgesic and anti-inflammatory potential of lupeol isolated from Indian traditional plant *Crateva adansonii* act as cheapest sources for multi-target agent with immense analgesic and anti-inflammatory potentials.

## Data Availability

The datasets used and analyzed during the current study available from the corresponding author on reasonable request.

## Abbreviations

CE: Chloroform extract
CMC: Carboxy methyl cellulose
ME: Methanolic extract
LF: Lupeol fraction
IL: Interleukin
IFN-γ: Interferon gamma
nAChR: Nicotinic acetylcholine receptor
COX-2: Cycloxygenase-2
MCP-1: Monocyte chemoattractant protein-1
RANTES: Regulated on activation, normal T cell expressed and secreted
MIP: Macrophage inflammatory protein
INDO: Indomethacin
MPO: Myeloperoxidase
PGE2: Prostaglandin E_2_
